# New Composite Materials Made from Rigid/Flexible Polyurethane Foams with Fir Sawdust: Acoustic and Thermal Behavior

**DOI:** 10.3390/polym14173643

**Published:** 2022-09-02

**Authors:** Ancuța-Elena Tiuc, Simona Ioana Borlea (Mureșan), Ovidiu Nemeș, Horațiu Vermeșan, Ovidiu Vasile, Florin Popa, Ramona Pințoi

**Affiliations:** 1 Department Environmental Engineering and Sustainable Development Entrepreneurship, Technical University of Cluj-Napoca, 400641 Cluj-Napoca, Romania; 2 Department of Mechanics, University Politehnica of Bucharest, 060042 Bucharest, Romania; 3 Department of Materials Science and Engineering, Technical University of Cluj-Napoca, 400641 Cluj-Napoca, Romania; 4 Department of Applied Mechanics and Civil Construction, University of Craiova, 200512 Craiova, Romania; 5 Research Institute for Construction Equipment and Technology—ICECON S.A., 060042 Bucharest, Romania

**Keywords:** wood fibers, sustainability, rigid and flexible polyurethane foam, morphological structure, acoustic absorbers, thermal conductivity

## Abstract

The aim of this work is to obtain new materials with improved sound absorbing and thermal properties, using rigid or flexible polyurethane foam reinforced with recycled fir sawdust from wood processing as well as by optimizing their mixing ratio. In this respect, we prepared and characterized samples by mixing rigid polyurethane foam (RPUF)/flexible polyurethane foam (FPUF) with 0, 35, 40, 45, and 50 wt% fir sawdust (FS) with grains size larger than 2 mm. The samples were evaluated by cell morphology analysis, sound absorption, and thermal insulation performance. The obtained composite materials containing 50% sawdust have superior acoustic properties compared to those with 100% FPUF in the range of 420–1250 Hz. The addition of 35% and 50% FS in the FPUF matrix led to improved thermal insulation properties and decreased thermal insulation properties in the case of RPUF. The results show that the use of FS-based composites with the FPUF/RPUF matrix for sound absorption and thermal insulation applications is a desirable choice and could be applied as an alternative to conventional synthetic fiber-based materials and as a recycling method of waste wood.

## 1. Introduction

Noise pollution is a reality of our society as result of the accentuated industrial development, heaving multiple harmful effects which must be controlled by smart specific methods [1]. Noise pollution lowers people’s mood and sleep quality and raises the frequency of numerous diseases to potentially fatal levels [2]. In the last years, scientists focused on the development of environmentally friendly sound-absorbing materials, using secondary raw materials, which can be recovered from different industries, and at the same time, strive to continuously improve the sound absorption performance of the existing acoustic materials [3]. The design and development of new environmentally friendly materials, clean technologies, and low-cost recycling strategies have become topics of study [4]. Thus, the use of waste materials in the production of environmentally friendly sound absorbing materials would help to solve three important problems in environmental protection: reducing the amount of waste, reducing noise pollution, and protecting natural resources [5].

In the last two decades, there has been tremendous research development on the use of several types of natural fibers in the developing of new types of sound absorbing materials. Traditional acoustic materials have certain limitations in sound insulation and low frequency absorption [6,7,8,9]. Raw natural fibers (wood fibers, vegetal fibers, animal fibers) as well as recycled materials have been studied as substitutes for non-biodegradable materials and are considered as alternatives to conventional materials due to cost efficiency, good durability, light weight, biodegradability, and lower environmental impact [9,10,11,12].

Polyurethane (PU) foams are one of the most important polymers that are widely used in various industrial fields such as furniture [13], thermal insulation materials [14], automotive [15], acoustic sound absorbing and sound insulating materials [16], lightweight structural materials [17], and vibration damping materials [18] due to their low cost, low density, and easy manufacturing processes [19]. Polyurethane foams are cellular polymers that can be either rigid—with closed pores—or flexible—with open pores [20]. PU foams are prepared by the addition of polymerization between multifunctional alcohols and diisocyanates, thus resulting in varied materials with a wide range of properties depending on the reactants. Since the types of commercially available diisocyanates are limited, polyols are the key elements in determining the properties of polyurethane foam. Thus, the following polyols’ properties are chosen according to the final utility of the foam: high temperature resistance, fire retardancy [21,22,23,24], ultraviolet resistance, rigidity of the cellular structure ensuring certain mechanical properties, flexibility for application on concave surfaces, spray application, slab molding, which are just some of the versatile applications of polyurethane foam [25]. Flexible polyurethane foams (FPUF) can be used in both thermal insulation and sound absorption, as well as in other technological applications. FPUFs are porous materials capable of absorbing sound energy, making them appropriate for noise control. The air flowing at the surface and inside the pores of a porous material exposed to incident sound waves are rendered into vibration and lose some of their original energy [26] because the energy of the air flow is converted to heat due to thermal and viscous losses in the internal pore walls and tortuosity in the material [27]. Thus, the most important characteristic of flexible polyurethane foam is to have interconnected open pore cavities, and its cellular structure plays a significant role in the sound absorbing properties. Rigid polyurethane foams (RPUF) are highly cross-linked three-dimensional polymers with a closed-cell structure, which account for approximately 23% of all polyurethane production [28,29]. Due to their excellent sound insulating properties, low thermal conductivity, high weather resistance, good mechanical properties, and low density, they are commonly used in industries such as construction, refrigerators, furniture, and in the production of thermal insulation materials [30,31]. Current research trends are directing to the production of flexible and rigid polyurethane foams by using environmentally friendly raw materials, introducing renewable resources, and addressing the problem of production and waste management at the industrial process design level [32,33,34].

The increasing need to replace petrochemical feedstocks with renewable ones together with the need to lower production costs have emphasized the development of polymer-based composites with natural fillers [35]. Thus, bird feathers, kenaf cellulose fibers, coconut fiber, flax, sisal, eggshells, walnut shells, rice husk ash, textile waste, sawdust, polyurethane foam waste, recycled rubber granules, rice husk, rock wool fiber, sunflower press cake, and jute have been used as fillers in flexible or rigid polyurethane foam matrices [36,37,38,39,40,41,42,43,44]. According to literature, the addition of filler to the polyurethane foam matrix leads to noticeable changes in the properties of the final composite material (thermal conductivity, density, and foam morphology) even when low filler content is used. In this respect, sawdust as a filler is a clean, cheap, and readily available as a by-product of wood processing such as milling, drilling, sanding, and sawing [45]. The use of sawdust as a filler in polyurethane foams provides a new use of recycled wood fibers in the development of new composite materials with applications in acoustics [40].

The present study aims to develop and optimize new composite materials based on polyurethane foams, using fir sawdust as a filler as well as to identify the correlation between the percentage of sawdust and the internal structure, acoustic and thermal properties of the composite material. The main goal of this research was to optimize the percentage of sawdust added in the rigid and flexible polyurethane foam matrix to obtain composite materials with better properties. An important novelty of this work is the simultaneous and comparative study of the two types of polyurethane foam structures under the same conditions. Thus, eight new composite materials with varying percentages of sawdust (35%, 40%, 45%, and 50%) for each type of structure (with closed and open pores) were developed and studied. The results obtained were compared with those of pure polyurethane foams, measured in the laboratory under the same conditions, and it was shown that the presence of sawdust in the rigid polyurethane foam matrix improves the sound absorbing properties and more in the flexible one improves the thermal insulation properties.

## 2. Materials and Methods

### 2.1. Materials

**Figure 1:**
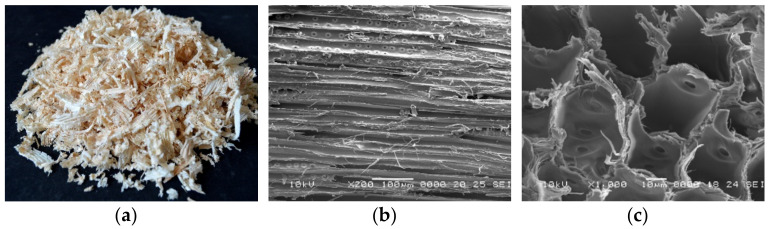
Fir sawdust used for composite plates: (**a**) overall aspect of fir sawdust; SEM images with a magnification of (**b**) ×200 and (**c**) ×1000.

To obtain sound-absorbing materials with superior properties, we must consider the nature, size, and proportion of the reinforcing material, the percentage and nature of the binder and finally, the manufacturing technology. The fir sawdust (FS) used to produce the composite materials (Figure 1a) was purchased from a solid wood furniture company. The main characteristics of the used sawdust are over 2 mm size, 10.1% moisture content, and 0.033 g/cm^3^ density. Following the previous research and from the literature, it was decided to sort the sawdust by grains size and to use only sawdust larger than 2 mm [40] because with smaller size sawdust, the specific surface area is larger and the binder absorption occurs before expansion, and thus the binder percentage used must be higher in order to obtain homogeneous materials. 

Knowing the wood structure is important not only for understanding its physical, mechanical, thermal, acoustic, electrical, and technological properties, but also for establishing different wood processing technologies. The microscopic analysis of wood reveals long tubular cells joined together by connecting points, which form the conductive tissue for the diffusion of water and mineral substances and can take up distorted mechanical stresses as a resistance tissue. Figure 1b,c shows the regular shape of the fibers, laid longitudinally with well-defined honeycomb capillary sections.

The moisture content of fir sawdust used in composite materials based on polyurethane foams is important due to the mixture’s sensitivity to water and/or moisture during reaction with isocyanates. The binder used in composites forms the bond between the reinforcement material, and it influences the physical, mechanical, and acoustic properties and establishes the final structure of the composite.

To produce composite materials with sound-absorbing properties based on fir sawdust, we used two bicomponent polyurethane foam systems as a binder, one to obtain a material with flexible structure—with open pores, and the other for a material with rigid structure—with closed pores. The two polyurethane foam systems are commercial products (produced by BASF). Flexible polyurethane foam (FPUF) Elastopor H 2400/5 was used to obtain composite materials with a flexible cellular structure with open pores with sound absorption properties. Rigid polyurethane foam (RPUF) Elastopor H 1221/41 was used to obtain composite materials with a rigid cellular structure with closed pores. Both systems have two components (A and B) where component A is a formed polyol. For the Elastopor H 2400/5 system, component A (density: 1.04 g/cm^3^, at 20 °C) is a mixture based on polyether polyol, catalyst, and blowing agent (water), and the Elastopor H 1221/41 system (density: 1.09 g/cm^3^, at 20 °C) contains polyol, catalyst, stabilizer, flame retardant, and blowing agent. Component B is polymeric diphenylmethane 4, 4′ diisocyanate (pMDI with 1.24 g/cm^3^ density, at 20 °C), which is one of the most widely used isocyanates in the production of polyurethane foams. pMDI is common to both systems, while the mixing ratio differs. The mixing ratio for Elastopor H 2400/5 is A:B=100:110 and the mixing ratio for Elastopor H 1221/41 is A:B=100:150. The two polyurethane foam systems contain no volatile organic compounds.

### 2.2. Preparation of the New Composite Materials

**Figure 2:**
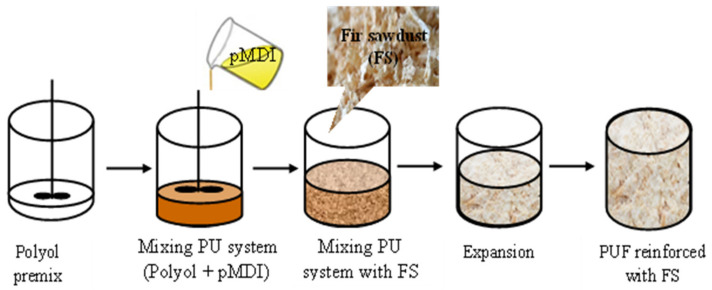
Schematic procedure of synthesis of FS reinforced PUF.

The production of the composite materials involved several steps, which were the same for both polyurethane foam systems (FPUF and RPUF) (Figure 2). The components of the polyurethane foam (PUF) were weighed and then mixed. Component A was poured into a polypropylene container and mixed energetically with component B at a weight ratio of 100:110 for FPUF and of 100:150 for RPUF. Pine sawdust was weighed and added to the PUF mixture at a weight ratio shown in Table 1 and mixed at 2000 rpm for 12 s for the flexible system and for 18 s for the rigid system. The mixture was quickly poured into the mold due to the high reaction speed. The mold was covered and left for 3–4 h to complete the chemical reaction and to achieve dimensional stability. After that, the material was removed from the mold [46].

Several experimental tests were performed to establish the optimal percentage of polyurethane foam necessary to obtain homogeneous and easy to handle plates, which would constitute the basis of study to analyze morphology, acoustic and thermal performance. Thus, the new composite mixtures contained 35%, 40%, 45%, 50% fir sawdust. To study the influence of material thickness on acoustic properties, materials with thicknesses of 40 mm and 60 mm, respectively, were prepared. Sixteen sample plates were prepared with different compositions (see Table 1). The coding and composition of the plates prepared is shown in Table 1. Apparent density of composite materials was determined according to EN ISO 845:2009 for 40 × 40 × 40 mm plates. The linear dimensions of the plates were determined based on the requirements of standard [47], the plates thickness was measured in parallel direction to the foam rise. 

**Figure 3:**
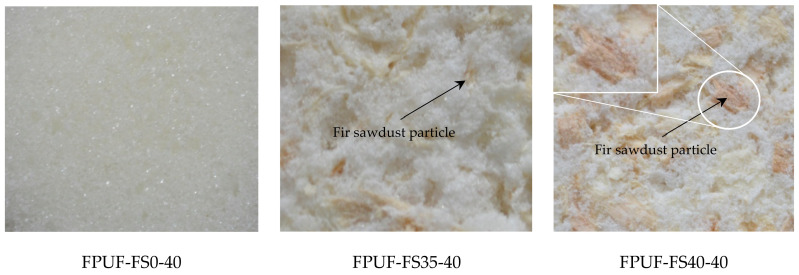

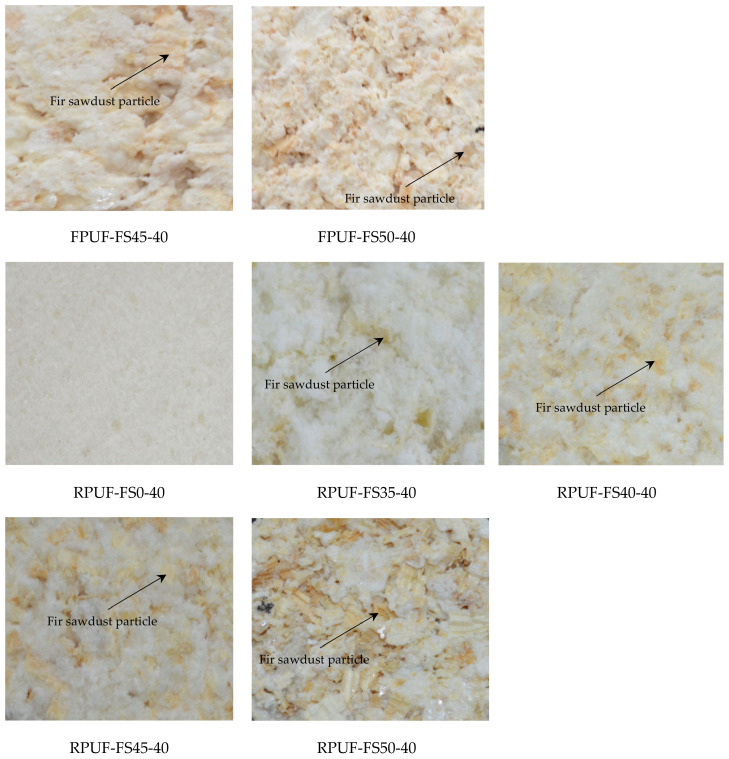
Surface images of the obtained plates.

Ten new composite materials were prepared and characterized within this research. For five mixtures, the following were used: flexible polyurethane foam (FPUF) as binder in plates 1, 3, 5, 7, and 8, with varying the binder percentage (100%, 65%, 60%, 55%, and 50%) and the thickness of the plates in three of the mixtures (samples 2, 4, and 6); and for the other five mixtures, the rigid polyurethane foam (RPUF) resulted in plates 9, 11, 13, 15, and 16, with varying the binder percentage (100%, 65%, 60%, 55%, and 50%) and plates thickness in three of the mixtures (plates 10, 12, and 14). Figure 3 show surface images of the 10 new composite mixtures; we can see the surface morphology depending on fir sawdust percentage.

### 2.3. Morphological Analysis

For the morphological analysis, a JEOL JSM 5600LV (Jeol, Tokyo, Japan) Scanning Electron Microscope (SEM) was used. The structure of the six new materials was analyzed by the SEM method to observe the influence of the percentage of sawdust and the type of polyurethane foam used. The morphology of materials with 0%, 35%, and 50% fir sawdust were analyzed for both systems: the flexible polyurethane foam. The samples’ morphology was studied in cross section of pure polyurethane foams and composites. The samples were cut perpendicular to the foaming direction. All the samples were analyzed using a secondary electron detector, at an accelerating voltage of 15 kV, under high vacuum. The samples were collected in a way to preserve the wood features and the images were recorded at low magnification to observe the important characteristics of the sample. To increase the resolution of the images, all the samples were coated with gold, using a DESK V sputter coater (Denton Vacuum, Moorestown, NJ, USA).

### 2.4. Acoustical Properties

The sound absorption coefficient was determined by the impedance tube method. Measurements were based on the transfer function method according to ISO 10534-2 [48]. The acoustic system for material testing consists of Brüel&Kjaer Type 4206-A medium impedance tube (Brüel&Kjær, Nærum, Denmark), two 4187 Brüel&Kjaer microphones (Brüel&Kjær, Nærum, Denmark), acoustic signal generator, PULSE 3560-B-030 analyzer (Brüel&Kjær, Nærum, Denmark), 2716 Brüel&Kjaer signal amplifier (Brüel&Kjær, Nærum, Denmark), and a PC for recording and processing the results, with the Brüel&Kjaer PULSE interface (Brüel&Kjær, Nærum, Denmark). For measuring the sound absorption coefficient of the composite materials, round samples with a diameter of 63.5 mm and a thickness of 40 mm and 60 mm, respectively, were prepared. The frequency range considered was 50–3150 Hz.

To analyze the new materials as comprehensively as possible in terms of sound absorption, 20 tests were carried out. Tests 1–16 were carried out under normal conditions of positioning the samples in the impedance tube and correspond to the 16 samples prepared, while tests 17–20 were performed by placing the samples 20 mm from the rigid wall of the impedance tube, resulting in the formation of an air gap between the material and the rigid wall, which influences the sound wave absorption process in certain frequency ranges (test 17 for FPUF-FS0-40, test 18 for FPUF-FS40-40, test 19 for RPUF-FS0-40, and test 20 for RPUF-FS40-40). Sample measurement conditions were as follows: air pressure 1005–1010 hPa; temperature 23.9–27.9 °C; relative air humidity 19–20%; sound velocity 345.51–347.83 m/s; air density 1.161–1.182 kg/m^3^; characteristic air impedance 403.8–408.5 Pa/(m/s).

### 2.5. Thermal Conductivity

Determination of thermal conductivity and thermal resistance was carried out according to EN 12667 [49], using a HLC A90 heat flow meter analyzer (HESTO Electronik GmbH, Steinbach, Germany), which has an active edge insulation. During the test, the direction of heat flow was upwards. Thermal conductivity measurements were conducted for 300 × 300 × 40 mm sized samples.

## 3. Results and Discussion

### 3.1. Morphological Characterization

**Figure 4:**
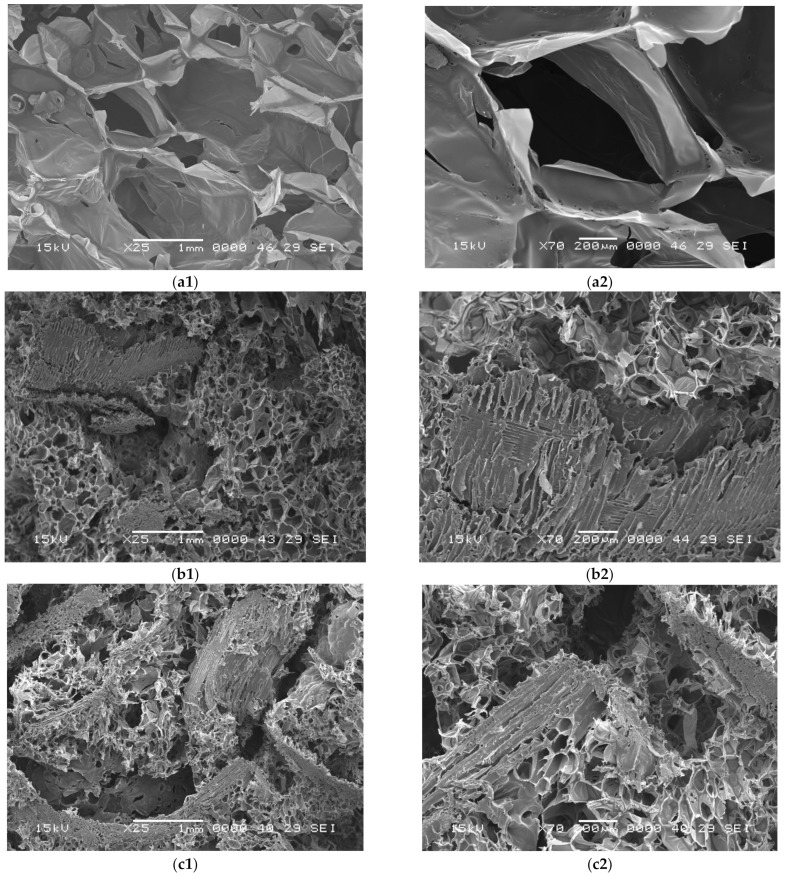
SEM images of obtained plates: (**a1**,**a2**) FPUF-FS0-40. (**b1**,**b2**) FPUF-FS35-40. (**c1**,**c2**) FPUF-FS50-40.

**Figure 5:**
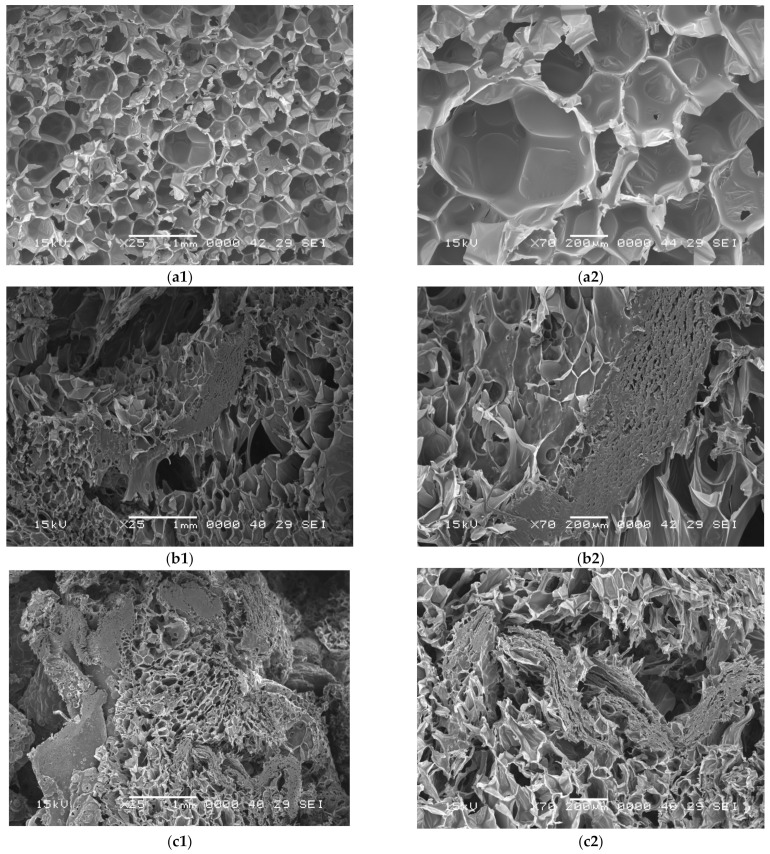
SEM images of obtained plates: (**a1,a2**) RPUF-FS0-40. (**b1,b2**) RPUF-FS35-40. (**c1,c2**) RPUF-FS50-40.

As seen in Figure 4 and Figure 5, the analysis of the SEM images reveal that the foam matrix adhered well to the wood fibers and the structure of the material differs according to the percentage and type of polyurethane foam. SEM images show that all samples have a non-uniform open or closed cell structure with spherical pores. As it can be seen, the addition of FS to the polyurethane matrix resulted in cells with more irregular and defective shapes and surfaces. 

The morphologies of the polyurethane foam pores, as shown in Figure 4 and Figure 5, depend on the percentage of sawdust added to the foam matrix, which in turn affects the expansion reaction [43,50]. Almost all pores of composite polyurethane foams were connected to adjacent pores due to the internal structure of the sawdust (longitudinal, regular shape of the fibers, well defined gofer shaped sections of capillaries Figure 1b,c) or by the distance between sawdust grains. The inter-connection of pores was positively associated with the amount of sawdust, especially when 50% sawdust was added into the polyurethane foam, as shown in Figure 4(c1,c2) and Figure 5(c1,c2).

Pore characteristics such as average pore size, number, type (open, semi-open, closed), and internal communication influence the acoustic and thermal insulation quality of the porous composite materials obtained. According to literature in most cases, there is a negative association between filler content and pore size, i.e., smaller pores are formed [51]. Increased viscosity of the polymer matrix, which is related to the addition of wood fibers, causes the decomposition of closed cells and conversion of open cells [52].

As seen in Figure 4, the microscopic structure of the 100% flexible polyurethane foam plates (Figure 4(a1,a2)) is predominantly with open and semi-open pores, while in composite materials, the presence of fir sawdust changed the pore type—resulting in more closed pores in the flexible polyurethane foam structure, connecting the sawdust parts, mainly due to the incomplete expansion (polymer expansion is partially affected due to increased mixture viscosity, leading to smaller pore formation [40,53])—and a part of the component was absorbed into the sawdust. However, what is important to note is the existence of adjacent open pores due to the presence of sawdust (Figure 4(b1,b2) and Figure 4(c1,c2)). Closed pores are mainly formed at the interface between the sawdust fibers and the polyurethane foam.

As seen Figure 5(a1,a2), the internal cellular structure of 100% rigid polyurethane foam plates has mostly closed pores. The addition of 35% (Figure 5(b1,b2)) and 50% (Figure 5(c1,c2)) fir sawdust leads to adjacent pores within the sawdust internal structure (longitudinal, regular shape of the fibers, well defined gofer shaped sections of capillaries) and increased distance between sawdust grains when the percentage is higher—thus open pores appear, which changes the material properties. Due to the increased viscosity in the composite materials as a result of the addition of a filler in the polyurethane foam matrix, the expansion process is no longer the same as in pure foam [44,54,55]. According to other research [56,57], sawdust acts as nucleation sites for cell formation. Thus, a larger number of cells start to form at the same time so there will be less gas for cell growth, resulting in smaller cell sizes, and there is less space to form open cells—when discussing flexible foams. Similar trends were observed in the study [58], where the authors used salinized and non-treated plum stone fillers in the rigid polyurethane foam matrix.

### 3.2. Sound Absorbtion Coefficients

In general, sound waves lead to the vibration of cell walls and air inside cavities and thus sound energy can be dissipated by damping the vibration of cavity walls and air [59,60,61]. Sound absorption performance can be improved due to sound energy reduction by increasing cell wall stiffness [62,63] and by interconnectivity, number, size, and type of pores. These are important parameters in the sound wave absorption mechanics inside a porous material, because the travel of sound waves inside the porous framework is essential to dissipate sound energy from visco-thermal couplings [64]. Decreasing the pores size in the open pore structure generally leads to an increase in airflow resistance and hence, sound absorption performance [25,65]. Increased interconnectivity in the porous material that can provide irregular transmission paths for sound waves favors sound absorption at lower frequencies [26].

**Figure 6:**
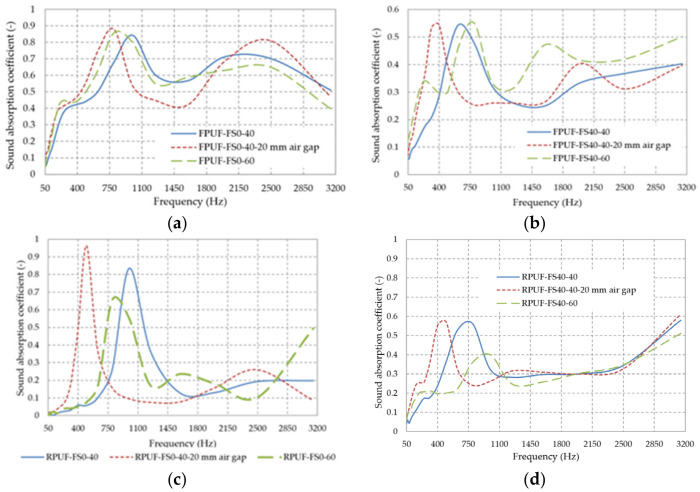
Sound absorption coefficient variation with: (**a**) thickness and position of flexible polyurethane foam samples, (**b**) thickness and position of flexible polyurethane foam samples with 40% fir sawdust, (**c**) thickness and position of rigid polyurethane foam plates, and (**d**) thickness and position of rigid polyurethane foam samples with 40% fir sawdust.

**Figure 7:**
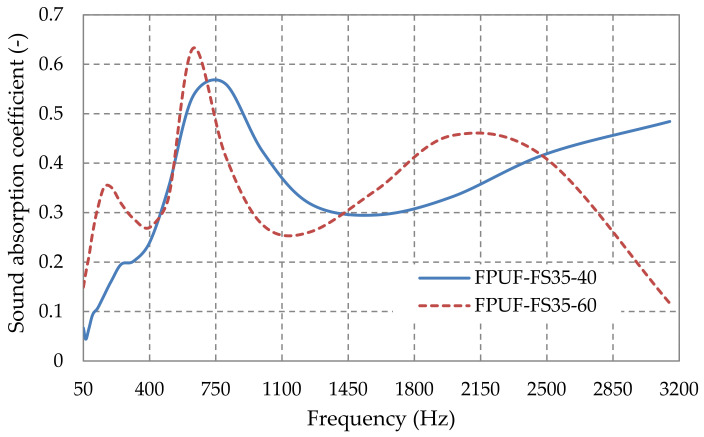
Sound absorption coefficient variation with plates thickness from flexible polyurethane foam with 35% fir sawdust.

**Figure 8:**
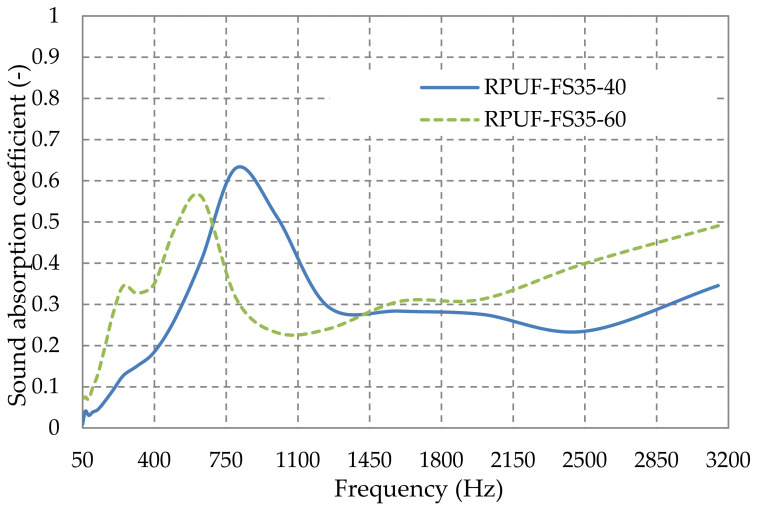
Sound absorption coefficient variation with plates thickness from rigid polyurethane foam with 35% fir sawdust.

**Figure 9:**
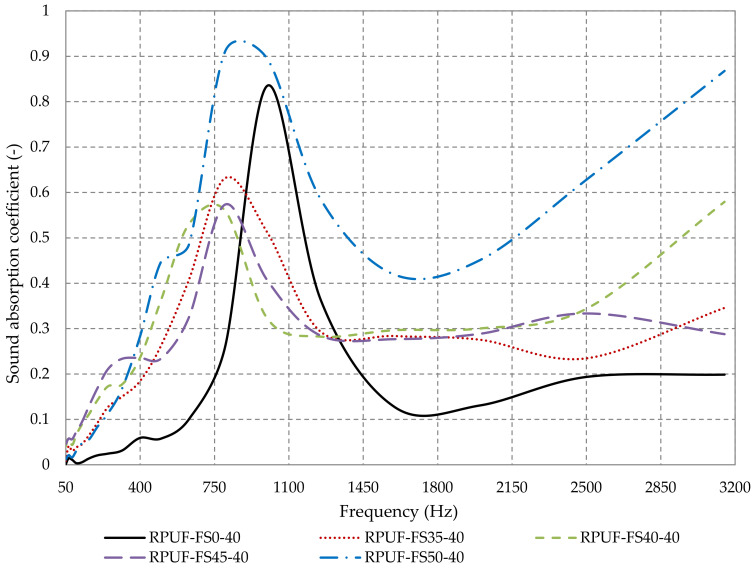
Sound absorption coefficient variation with the fir sawdust percentage in rigid polyurethane foam samples.

**Figure 10:**
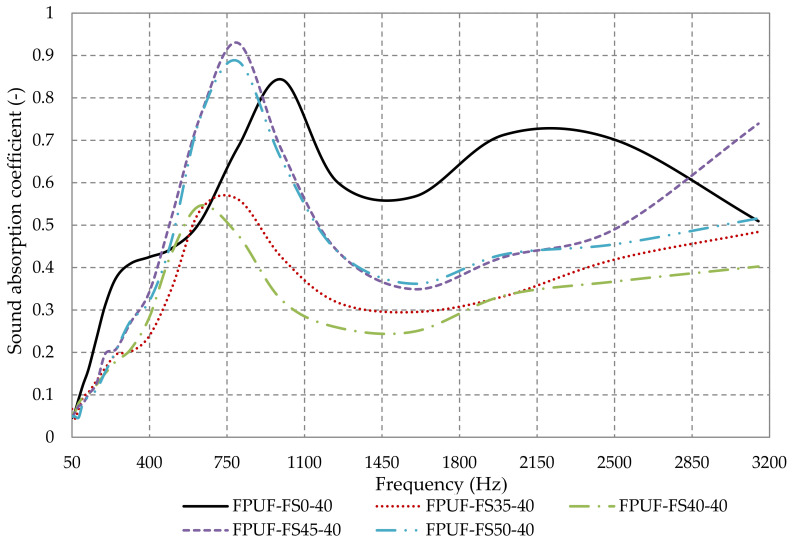
Sound absorption coefficient variation with the fir sawdust percentage in flexible polyurethane foam samples.

**Figure 11:**
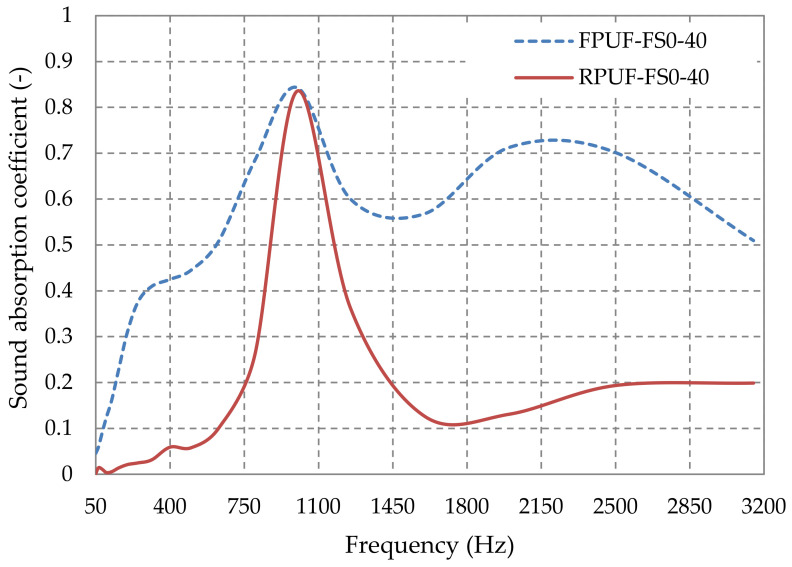
Sound-absorption coefficient variation with the type of polyurethane foam.

**Figure 12:**
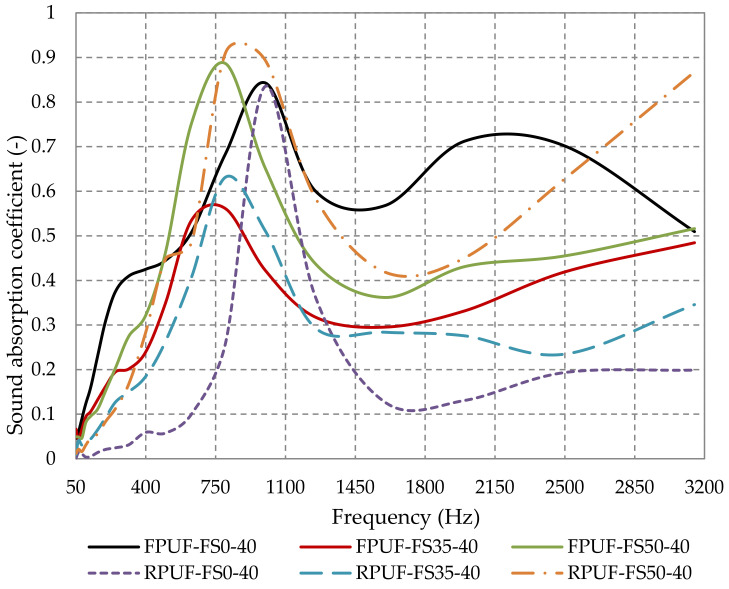
Sound-absorption coefficient variation with the type of polyurethane foam and the fir sawdust percentage.

**Figure 13:**
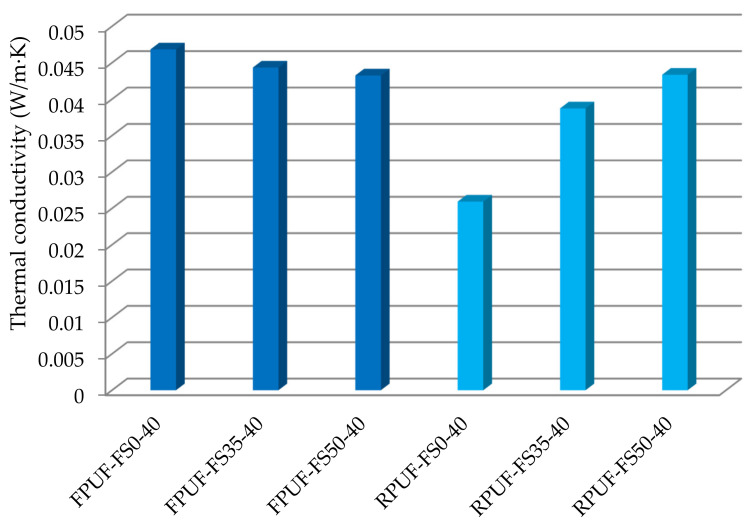
Thermal conductivity of obtained plates.

Sound absorption capacity is influenced by the material thickness, the placement of the material (leaving a space between the material and the rigid wall), and the internal structure of the material (the percentage of fir sawdust and the type of polyurethane foam used). Based on the results obtained in this research, the influence of these parameters is evidenced and compared with the literature. If the absorption coefficient has high values over a wide frequency range, the material is considered having good sound absorbing properties. The normal incidence sound absorption coefficients (α) of materials are a function of the sound frequency (f), as shown in Figure 6, Figure 7, Figure 8, Figure 9, Figure 10, Figure 11, Figure 12 and Figure 13.

#### 3.2.1. Influence of Material Thickness and Placement on Acoustic Properties

Figure 6 show the effect on the sound absorption coefficient of the material thickness and its placement. The influence of material placement is shown by placing samples in the impedance tube at 20 mm from the rigid wall of the impedance tube. The distance between samples and the wall results in improved sound absorption properties, especially for low frequencies [3,40]. Various research on the influence of thickness on sound absorption concluded that by increasing thickness, the value of sound absorption coefficient increases at low frequencies, while this effect is not observed at high frequency [66,67].

Material thickness is one of the most important characteristics influencing sound absorption performance. Changing thickness changes other parameters, including density and porosity [68]. Since these parameters (thickness and air layer) improve absorption in the low frequency range, we analyzed their influence, and we suggest that in certain frequency ranges, it is more efficient to use a thinner material in combination with an air layer rather than a thicker material, which is useful when having load limitations of an existing construction or to reduce costs.

As shown in Figure 6a, the sound absorption coefficient at frequencies bellow 800 Hz is the best for flexible polyurethane foam with a thickness of 40 mm, placed at 20 mm from the rigid wall, when compared to FPUF-FS0-60. The sound wave passes through the sample, inside the material it travels a path due to the open pores, through the air gap (the space between the sample and the rigid wall of the impedance tube), is reflected by the rigid wall, and then returns through the air gap and through the material [69]. As seen in Figure 6b, the peak of the sound absorption coefficient shifted to the lower frequency range when an air gap of 20 mm was present, for flexible foam-based composites with 40% FS. The stiffness of the material was reduced, the open pores work as an acoustic mass, while the air gap acts as acoustic spring, resulting in a resonance between the mass and the spring [30]. At frequencies over 700 Hz, the use of a 60 mm thick composite material is recommended. The peak of the sound absorption coefficient shifted towards the frequency range below 600 Hz when using a 20 mm air gap between the rigid polyurethane foam and the impedance tube wall, as shown in Figure 6c. What is notable in the case of rigid foams is that the narrow frequency range remains even when using an air layer, and only the peak shifts. As it can be seen, increasing the thickness of the rigid foam from 40 mm to 60 mm improves absorption in the frequency range 50–800 Hz, 1400–2200 Hz, and 2600–3150 Hz. In the case of composites with 40% sawdust in the rigid polyurethane foam matrix, the peak of the sound absorption coefficient shifted to lower frequencies when using a 20 mm air gap—from 750 Hz for RPUF-FS40-40, to 430 Hz for the RPUF-FS40-40-20 mm air gap, as seen in Figure 6d.

Figure 7 shows the sound absorption coefficient variation with frequency by varying the thickness of the composite material with 35% sawdust in the flexible polyurethane foam matrix. As it can be seen, increasing the thickness results in an increase in sound absorption properties in the frequency range 50–700 Hz and 1400–2500 Hz.

Figure 8 shows the variation of the sound absorption coefficient with frequency, by varying the thickness of the composite material with 35% sawdust in the rigid polyurethane foam matrix. Here, again, we observe the improvement of the sound absorbing properties with material thickness, at low frequencies, as reported in the literature [68].

#### 3.2.2. Influence of Sawdust Percentage on Sound Absorption Coefficient

Theoretically, the sound absorbing properties of a porous material are mainly influenced by the size and structure of the pore cells [70]. Large porous cells and interconnected cells favor sound absorption at low frequencies. The presence of a filler, FS in our case, modifies the open or closed porosity of the polyurethane foam, and the interconnection of the pores and the formation of adjacent pores, as confirmed by the morphological analysis performed (Figure 4 and Figure 5). It reduces the size and volume fraction of the air pores and makes the air passages more convoluted and much narrower, thus making the sound wave travel a longer path until it is reflected, and thus reducing its energy. This correlation was observed also in other research [71].

As seen in Figure 9, in the case of closed-pore rigid polyurethane foam composite materials, fir sawdust (35%-RPUF-FS35-40, 40%-RPUF-FS40-40 and 45%-RPUF-FS45-40) is shown to improve the sound absorption coefficient at frequencies below 840 Hz and above 1400 Hz, as compared to 100% RPUF material. The 50% pine sawdust composite material, RPUF-FS50-40, has better absorption coefficient values over the whole frequency range considered, when compared to the 100% RPUF material. As we can see in the SEM images, the increase of the percentage of fir sawdust led to the increase of the open porosity and interconnections, due to the internal structure of the sawdust and the larger size of the sawdust grains, which allowed the formation of pores between the grains. According to previous research, when the sawdust particles size is below 2 mm, a compact bond is formed between its grains, which finally leads to the decrease of the porosity and implicitly of the sound absorption properties of the material [40].

As can be seen in Figure 9, another important aspect is that the rigid polyurethane foam, RPUF-FS0-40, has a very narrow peak at low frequencies, which is mainly due to a reduced interconnected porosity at the surface of the material [72]. In the case of composite materials, there is a broadening of the frequencies range where the sound absorbing properties are improved. The RPUF-FS50-40 material shows an even significant improvement of sound absorption properties, reaching a minimum absorption coefficient of 0.4 in a wide range of frequencies of 400÷3150 Hz, when compared to RPUF-FS0-40, which reached a minimum of 0.1. Thus, the use of composite materials based on rigid polyurethane foam improved the sound absorption properties over a wide range of frequencies compared to 100% RPUF material.

Figure 10 shows the influence of the percentage of fir sawdust used in composite materials, on the sound absorption coefficient. The materials studied were made of open-pore flexible polyurethane foam and sawdust in the proportion of 0%, 35%, 40%, 45%, and 50% (FPUF-FS0-40, FPUF-FS35-40, FPUF-FS40-40, FPUF-FS45-40, and FPUF-FS50-40). In the case of materials with a sawdust percentage of 50% and 45%, the sound absorption coefficient improves in the frequency range 450–900 Hz, when compared to the 100% flexible polyurethane foam, while at frequencies higher than 900 Hz, the sound absorption properties decrease. In the case of materials with 40% and 35% fir sawdust, the sound absorption coefficient is lower over almost the entire frequency range considered, when compared to the 100% polyurethane foam material, except in the frequency range 400–600 Hz, where the sound absorption coefficient has better values.

In the case of flexible polyurethane foam, vibroacoustic energy is transported both through the air in the pores and through the solid materials of the frame itself. Amplitude and phase differences convert part of the mechanical–acoustic energy into heat, mainly due to viscoelastic and viscoacoustic phenomena in the solid frame and at the interface between the solid frame and the pore air. Thus, the sound absorption efficiency in open-pore flexible foams, FPUF-FS0-40, is composed by a low sound reflection and is mainly based on sound dissipation [73]. When sawdust is used, the surface tension between polymer and gas is reduced, which leads to a decrease in the free energy required for nucleation and consequently increases the nucleation rate [43,74,75] and thus, the complete formation of open cells specific to flexible foam does not occur. This can also be seen in the morphological analysis of FPUF-based composite materials. Incomplete formation of open cells negatively influences the composite materials absorption process when compared to FPUF-FS0-40, according to the results shown in Figure 11. Increasing the density of the composite foam as the FS percentage is increased also seems to have an important effect on the sound absorption coefficient, since a high apparent density in a foam means less void space to dissipate sound as heat energy [26].

#### 3.2.3. Influence of the Type of Polyurethane Foam on Sound Absorption Coefficient

The sound absorption mechanism in porous materials is strongly corelated with the number, size, and type of pores. Sound must enter the porous material, which means that there should be enough open pores at the surface of the material to allow it [5]. Much of the energy dissipated within a porous material result from the relative motion of the solid framework and interstitial fluid. For this process to work, there must be continuous pathways through the material, i.e., the pores of the material must be connected [6]. Therefore, closed-cell materials have a lower noise reduction capability (Figure 11). Figure 11 shows the influence of the internal structure of a porous material, i.e., whether the pores are open or closed, on the sound-absorbing properties—the variation of the sound absorption coefficient with frequency for materials made of 100% polyurethane foam is shown. The material with 100% open-pore polyurethane foam has higher sound absorption coefficient values over the whole frequency range considered. However, as it can be seen, the maximum absorption reached is 0.84 for the open-pore material and 0.83 for the closed-pore material, at 1000 Hz.

The ability of rigid polyurethane foams to absorb noise at low frequencies is relatively low [76]. However, as seen in Figure 12, the sound-absorption coefficient can be improved by adding fir sawdust. As seen in Figure 12, overall, flexible polyurethane foam materials have better sound-absorbing properties than rigid polyurethane foam materials.

According to the literature, it is known that flexible polyurethane foam is one of the most widely used sound-absorbing materials due to its good sound-absorbing properties over wide frequency ranges [3,77]. Nevertheless, in the frequency range 420–1250 Hz, 50% sawdust composite materials (FPUF-FS50-40 and RPUF-FS50-40) have superior acoustic properties to 100% open-pore polyurethane foam. RPUF-FS50-40 has the highest peak of 0.91 at 800 Hz and shows the best sound-absorbing properties in the frequency range 740–1240 Hz.

### 3.3. Thermal Conductivity 

As it is well known, the thermal conductivity of polyurethane foams is highly dependent on the density of the foam, the morphological properties of the pores (closed, semi-closed or open pores), the conductivity of the gas mixture in the pores, the conductivity of the matrix polymer, the radiation between the cells, and the moisture content of the filler [78,79,80]. For this reason, the bulk densities of the materials prepared in this study were determined according to the relevant standard to correlate their thermal insulation performance and density, as seen in Table 1. To be able to explain the thermal conductivity and according to the internal morphology, the same mixtures were measured. Thus, morphological analysis was performed for 0%, 35%, and 50% pine sawdust content for both systems (flexible polyurethane foam FPUF-FS0-40, FPUF-FS35-40, FPUF-FS50-40, and rigid polyurethane foam RPUF-FS0-40, RPUF-FS35-40, and RPUF-FS50-40). Thermal conductivity is the property of materials to transmit through their mass the heat flow produced by the temperature difference occurring on opposite surfaces of building elements. The lower the coefficient of thermal conductivity, the higher the thermal resistance of the material, and therefore the better the material in terms of thermal insulation [79,81].

Thermal insulation performance of materials has a direct relation with the internal structure and decreases with increasing porosity [80,81]. The porosity of the new materials studied in this study depends on the type of polyurethane foam and the presence of fir sawdust (it has a porous structure and a large specific surface area [81]). Following the measurements performed and the processing of the resulting data, the values of the thermal conductivity coefficient (Figure 13) for the studied materials were obtained. The presence of fir sawdust in the flexible polyurethane foam matrix resulted in decreased thermal conductivity, i.e., improved insulation with the increase of FS percentage, when compared to 100% flexible foam FPUF-FS0-40, as seen in Figure 13. The presence of sawdust did not allow full expansion and formation of the open pore cellular structure, and thus the number of open pores was reduced. Another important factor that favoured the improvement of the insulation properties was the increase of the apparent density of the composite materials: FPUF-FS0-40—0.026 g/cm^3^, FPUF-FS35-40—0.075 g/cm^3^, and FPUF-FS50-40—0.116 g/cm^3^. These results are in accord with the literature data [56,80,81]. By improving the thermal insulation properties by adding FS, which led to the closing of the pores in the case of FPUF, it could be observed that the acoustic absorption properties decreased because, in the case of absorption, we need open pores.

The influence of the presence of FS in the rigid polyurethane foam matrix is shown in Figure 13. The thermal insulation properties decrease with increasing sawdust percentage, exactly the opposite as in the case of FPUF matrix composites. These results may be related to changes in the morphological structure of the foams, which is the increasing of open porosity mainly due to the porosity of the sawdust and adjacent pores, according to the morphological analysis. The decrease in the thermal insulation properties by adding FS in the RPUF matrix due to the increase in open porosity led to the improvement of the acoustic absorption properties. It is known that rigid polyurethane foam is one of the most widely used materials with the best thermal insulating properties [14]. RPUF-FS0-40 has a thermal conductivity of 0.0259 W/m·K compared to expanded polystyrene (0.044 W/m·K) and glass wool (0.055 W/m·K) [82]. Even though RPUF-FS35-40 and RPUF-FS50-40 composites have higher thermal conductivity values compared to rigid foam, 100% of their thermal insulation properties are good when compared to other materials on the market.

By adding a percentage of 50% fir sawdust into the matrix of FPUF and RPUF, two new composites materials result with approximately the same thermal conductivity (FPUF-FS50-40 with 0.0432 W/m·K and RPUF-FS50-40 with 0.0433 W/m·K) although the initial foam matrix differs for the two materials, hence we can see the importance of the filler structure when added in high percentages in the composite material. With the addition of 50% sawdust, the thermal insulating properties of FPUF and the sound-absorbing properties of RPUF were considerably improved. Thus, these composite materials show a superior use of wood waste and conservation of resources needed to obtain polyurethane foams.

## 4. Conclusions

The study presented in this work proposes to develop new composite materials by valorization of fir sawdust, as filler, in a rigid/flexible polyurethane foam matrix with the aim to improve the sound-absorbing and thermal insulating properties of the materials, at a low cost and with the lowest possible environmental impact. The use of pine sawdust in the production of sound-absorbing materials is a major step in reducing the amount of wood waste and superior recovery of it.

Sixteen composite materials plates were obtained. Eight new composite material mixtures with varying percentages of sawdust (35%, 40%, 45%, and 50%) in a closed pore and open pore foam structure were developed and analyzed.

The performance of new obtained composite materials depends on:

- the morphological structure,
- the percentage of sawdust in the matrix,
- the type of the matrix,
- the thickness of the plate,
- the position of the material in the structure.

According to obtained results, increasing the FS in FPUF can partially inhibit the uniform expansion of foam cells, resulting in irregular cell structure and reducing the number of open pores while the presence of FS in RPUF lead to a more open porosity. The sound absorption of closed-pore rigid composite materials improved for the frequency range below 700 Hz with increasing thickness of the plates, and the presence of a 20 mm air gap. New composite materials FPUF-FS50-40 and RPUF-FS50-40 (50% fir sawdust) have superior acoustic properties when compared to the 100% open-pore polyurethane foam in the frequency range of 420–1250 Hz. The presence of 35% and 50% fir sawdust in the FPUF matrix resulted in improved thermal insulating properties with the increase in the percentage of FS, in comparison with the flexible foam without filler. In the case of RPUF, the thermal insulation properties decreased with the increase of sawdust percentage. The thermal conductivity of FPUF-FS50-40 has 0.0432 W/m·K close to RPUF-FS50-40 with 0.0433 W/m·K, even if the matrix in these materials was different. Thus, the importance of the filler structure present in high percentages in the composite material is emphasized. By adding 50% sawdust, the thermal insulating properties of FPUF and the sound-absorbing properties of RPUF have been improved.

As a global conclusion, the new composite materials plates can be used as acoustic barriers and as construction materials to reduce or insulate noise as well as a thermal insulator. The characterization of these materials in terms of mechanical properties, water absorption, thermal stability, as well as the development of acoustic panels that would use these materials, are in progress.

## Figures and Tables

**Table 1:** Sample plates codification.

Sample No.	Code	Fir Sawdust (wt%)	Binder Type	Thickness (mm)	Apparent Density (g/cm^3^)
1	FPUF-FS0-40	0	Flexible polyurethane foam	40	0.026
2	FPUF-FS0-60	0		60	0.026
3	FPUF-FS35-40	35		40	0.075
4	FPUF-FS35-60	35		60	0.075
5	FPUF-FS40-40	40		40	0.094
6	FPUF-FS40-60	40		60	0.094
7	FPUF-FS45-40	45		40	0.108
8	FPUF-FS50-40	50		40	0.116
9	RPUF-FS0-40	0	Rigid polyurethane foam	40	0.034
10	RPUF-FS0-60	0		60	0.034
11	RPUF-FS35-40	35		40	0.089
12	RPUF-FS35-60	35		60	0.089
13	RPUF-FS40-40	40		40	0.102
14	RPUF-FS40-60	40		60	0.102
15	RPUF-FS45-40	45		40	0.117
16	RPUF-FS50-40	50		40	0.125

## Data Availability

The data presented in this study are available on request from the corresponding authors.
